# Quality-adjusted life years among people who inject drugs in a needle syringe program in Sweden

**DOI:** 10.1007/s11136-022-03209-9

**Published:** 2022-08-22

**Authors:** Martin Kåberg, Sofie Larsson, Jakob Bergström, Anders Hammarberg

**Affiliations:** 1 Department of Global Public Health, Karolinska Institutet, 171 77 Stockholm, Sweden; 2 Stockholm Centre for Dependency Disorders, Stockholm Needle Exchange, Stockholm, Sweden; 3 Department of Public Health Analysis and Data Management, Public Health Agency of Sweden, Stockholm, Sweden; 4 Centre for Psychiatry Research, Department of Clinical Neuroscience, Karolinska Institutet, Stockholm, Sweden; 5 Stockholm Centre for Dependency Disorders, Stockholm Health Care Services, Region Stockholm, Stockholm, Sweden

**Keywords:** HRQoL, QALY, EQ-5D, SF-6D, People who inject drugs, Needle syringe program, Harm reduction

## Abstract

**Purpose:**

Needle syringe programs (NSP) significantly reduce risk behavior and HIV and hepatitis transmission in people who inject drugs (PWID). However, PWID are underrepresented in studies on health-related quality of life (HRQoL), representing a barrier to evaluate effects of public health and preventive measures related to injecting drug use. In this study, we investigate how well the two questionnaires EQ-5D-3L and SF-6D measure health in PWID. We also estimate HRQoL in the PWID population.

**Method:**

Data on demographics, injection drug use, HIV, hepatitis status, and self-reported HRQoL were collected from 550 PWID enrolled in the Stockholm NSP at enrollment and at 6-, 12-, and 24-month follow-up. Self-rated HRQoL was measured as QALY, using EQ-5D-3L and the SF-6D. Item response theory (IRT) was used to evaluate which of the two instruments that measure health most accurately in this population. Regression analysis was used to estimate population-specific QALYs.

**Results:**

The IRT analysis showed that SF-6D was better suited to measure health in PWID. More specifically, SF-6D to a larger extent discriminated between persons regardless of their health status, while EQ-5D was more suitable to detect persons with poorer health. Self-rated HRQoL showed that average QALY was lower among PWID compared to the general Swedish population. However, a general increase in self-reported health was noted over time among participants.

**Conclusion:**

This study increase knowledge of what instruments are most suitable to measure health among PWID. This is of great importance when evaluating effects of public health and preventive measures in the PWID population.

## Introduction

There are an estimated 275 million people worldwide using drugs, of which 36 million fulfill the criteria for substance use disorder (SUD). Among those, approximately 11 million were people who inject drugs (PWID) [1]. In 2013, the global burden of disease related to previous exposure to HIV, hepatitis B (HBV), and hepatitis C (HCV) among PWID was high and accounted for more than 10 million disability-adjusted life years (DALYs) [2]. In 2016, 32 million DALYs were attributed to drug use as a risk factor, including SUD, infectious diseases, liver cirrhosis, and self-harm as the main causes [3].

Needle syringe programs (NSP), significantly decrease both risk behavior and HIV and HCV transmission in PWID [4–7]. However, due to marginalization and stigmatization, PWID are often subjected to social and economic barriers for accessing public health and preventive measures of adverse injection drug use-related health consequences [8, 9]. Consequently, PWID have been underrepresented in studies on health-related quality of life (HRQoL). However, to make informed decisions on how to allocate resources in health care, it is important to have information on both health measures and resource use in different populations. Also, when prioritizing interventions aiming to increase health in PWID it is important to have accurate measures of health in this population. HRQoL is a measure often used in such considerations, e.g., regarding the cost effectiveness of health care interventions. Different instruments are used to describe and measure HRQoL, of which some are disease specific and some are generic, e.g., the EuroQol-5 Dimension (EQ-5D) and the Short Form-6 Dimensions (SF-6D) questionnaires [10].

In 2008 Vickerman et al. conducted a cost-effectiveness analysis (CEA) of NSP in Australia. Since no data on HRQoL for PWID existed, they assumed that quality-adjusted life year (QALY) weights for PWID were on average 90 percent of weights for the general population [11]. The same multiplicative factor has been used in other studies since people with SUD had a higher prevalence of comorbid psychiatric disorders [12, 13]. An updated CEA on NSP and opioid agonist therapy (OAT) on HCV transmission among PWID in 2017 refer to an arbitrary QALY weight of 84 percent, applied from a study by Martin et al. in 2016 [14, 15].

McDonald et al. also studied quality of life in PWID with regards to HCV infection status [16]. They found that awareness of chronic HCV infection was associated with a decrease in HRQoL, but that there was no evidence of further reductions due to the infection itself. Their results also suggest that age, gender, and if ever homeless decrease HRQoL among PWID. Studies on HRQoL in OAT populations have associated OAT, employment, and amphetamine use with higher HRQoL, while older age, longer duration of opioid dependence, HCV and HIV infection, impaired physical and mental health, and psychopharmacological medication were associated with lower HRQoL [17, 18].

In CEA, HRQoL is often measured as QALYs since it combines quality of life with length of life into one outcome measure, which enables comparisons and prioritizations between interventions in different therapeutic areas. However, in studies focusing on health effects among PWID, other measures than QALY have also been applied. In 2013, Fischer et al. studied QoL in PWID enrolled in an NSP in Brisbane, Australia, using the World Health Organization Quality of Life (WHOQOL-BREF) instrument [19, 20], which is not transferable to QALY, but also measure environmental aspects of quality of life. In this study, it was found that PWID have poor health irrespective of socio-demographic characteristics, injecting patterns or HCV status. In addition, general health in PWID were below what was experienced in populations with disabling chronic diseases. Furthermore, HRQoL did not differ with regards to HCV infection status or between daily or occasional injectors [19]. In a Swedish report, investigating quality of life among people living with HIV, a significantly lower self-rated quality of life among people who used drugs was noted, especially among PWID [21].

To summarize, the literature on HRQoL in PWID is scarce. Furthermore, there are a variety of different instruments to assess HRQoL, not specifically targeting PWID. Measures of HRQoL in PWID are important when evaluating different interventions aiming at enhancing PWID’s health. It is thus essential to apply instruments for assessing the value of health interventions embracing mental well-being as well as physical functioning among PWID. The aim of this study was to assess how well EQ-5D and SF-6D measures health in PWID and to estimate HRQoL, measured in QALYs, in this population.

## Methods

### Study setting and inclusion

The Stockholm NSP offers sterile injection equipment, i.e., needles/syringes and paraphernalia (cookers/filters), and testing for HIV, HBV, and HCV at inclusion [22–24]. General counseling, treatment for infectious diseases, referrals to social services, and substance use clinics, including OAT, are provided. The NSP is organized by physicians and nurses specialized in infectious diseases and psychiatry/addiction medicine, counselors, and midwives.

This study was part of a larger study sample investigating NSP long-term effects among PWID in Stockholm, Sweden, 2013–2018 (*n = *1386–2860). In these study populations the mean age was 38.0–39.3 years; 23.2–24.0% were females; 77.3–80.2% Swedish born; 36.3–36.7% with independent living; 12.0–13.8% employed; 55.0–62.1% HCV positive; 4.9–6.7% HIV positive; 1.4–2.1% HBV positive; 9.1–17.6 on OAT; 43.3–44.3% using amphetamine; and 36.9–39.2% using heroin. [22–24].

Participants enrolled in the NSP between April 2013 and April 2015 who fulfilled the following criteria were eligible for inclusion in the study: (1) above 20 years of age and (2) active (i.e., current) injection drug use. At inclusion, participants were informed of the study in a written and oral form, had the possibility to ask further questions, and provided written informed consent.

At enrollment, demographic and related data were collected regarding age, country of birth, housing status, employment status, participation in OAT, frequency of injection drug use, and last drug injected. Data on last drug injected was collected at every visit, while other demographic data were updated every three to six months. Participants were also repeatedly screened for HIV, HBV, and HCV through venipuncture independent of symptoms every three to six months, and blood samples were analyzed at the Karolinska University Hospital laboratory.

### Quality of life measures

At enrollment in the study and at 6-, 12- and 24-month post-enrollment, self-rated HRQoL was measured as QALY, using the EuroQol-5-dimension, 3-level version (EQ-5D-3L) and Short Form 12 Health Survey (SF-12) questionnaires [25–27]. EQ-5D consist of five items (mobility, self-care, usual activities, pain/discomfort, and anxiety/depression) and was transformed into QALY using the value set by Dolan [28]. We used the version in which each item in EQ-5D contain three-level answers, resulting in a total of 243 possible unique health states. QALY weights can range from − 0,594 (worst health) to 1 (best health) when using the value set by Dolan [28].

The SF-12 instrument consists of 12 items. To estimate QALY weights from SF-12 we transformed answers to the SF-6D instrument using the standard algorithm by Brazier and Roberts [29]. The SF-6D instrument consists of six items (physical functioning, role limitation, social functioning, pain, mental health, and vitality). SF-6D questions are answered in 3, 4, or 5 levels, resulting in a total of 7500 different health states. QALY weights can range from 0.345 (worst health) to 1 (best health) when using the value set by Brazier and Roberts [29].

Demographic data were collected in conjunction with HRQoL measurement, while we considered current HIV, HBV, and HCV status to be the last known test result for these infections. Health status was not mutually exclusive, which means that one person could have more than one of these infections at the same time.

### Data analysis

In the first part of the analysis, we tested which of the instruments that were most relevant to measure health in the PWID population using item response theory (IRT), and in the second part we estimate the HRQoL (in QALY) using the most relevant instrument identified from part one of the analysis.

### Part 1: Analysis of instruments – Item response theory

Item response theory (IRT) is used to study unobservable characteristics, such as health, in different groups or settings [30]. The unobservable characteristic is often intuitively understood, e.g., we can understand “good health” or “bad health”; however, it cannot be directly measured, such as length or weight, since it often depends on several different aspects of the characteristic [31]. Instead, instruments with a collection of items or dimensions are used to indirectly measure the characteristic of interest. In our study, the unobservable characteristic is HRQoL, measured as QALY. We used IRT to analyze how each EQ-5D or SF-6D dimension (in IRT referred to as *item*), as well as the instruments as a hole (groups of items) relates to HRQoL (the unobservable characteristic).

For the IRT analysis, we had five items in the EQ-5D questionnaire and six items in the SF-6D questionnaire. An underlying assumption of an IRT model is that items within a questionnaire (instrument) only measure one dimension, i.e., a unidimensional characteristic, which was HRQoL in our study. To check the assumption of unidimensionality we performed a principal component analysis (PCA) prior to the IRT analysis [32].

To compare EQ-5D and SF-6D questionnaires a two-parameter IRT model for polytomous items (graded response model) was carried out. The two parameters estimated was (a) item discrimination, i.e., how well each item could differentiate among respondents at different health levels, and (b) item difficulty, i.e., how a respondent’s HRQoL affect the level of response in the different items. According to guidelines proposed by Baker [31], an item discrimination parameter above 1.35 is classified as high discrimination power. In the IRT analysis, standard errors were clustered at individual level to account for repeated measurements within the same individual.

We also assessed how well the instrument differentiates among respondents and at what ranges of health, by estimating the Test Information Function (TIF) for both EQ-5D and SF-6D. Range of health was measured as theta, with theta = 0 indicating average health. In addition, theta greater than zero equals health state worse than average, which means that health is decreasing with theta increasing [31] and the opposite for theta smaller than zero.

### Part 2: Analysis of HRQoL

Given the results in part one we used answers from the questionnaire (EQ-5D or SF-6D) that was considered most suitable to measure health in this population to analyze the effect of different personal characteristics on the estimated QALY weight. We used an OLS regression, with the following (full) model specification:

1 $$QALY=\alpha +{\beta }_{1}*Age+{\beta }_{2}*Female+{\beta }_{3}*Health\,status+{\beta }_{4}*Substance\,use+{\beta }_{5}*Employment+{\beta }_{6}*OST+ {\beta }_{7}*Independet\,living+{\beta }_{8}*Daily\,drug\,use+\varepsilon.$$

‘Age’ was categorized into 10-year age groups, ‘Female’ a binary variable that takes the value 1 if respondent is female and 0 otherwise. Health status was a vector of different binary variables (HIV, HBV, and HCV), which were not mutually exclusive, and ‘Substance use’ was a vector of binary variables of different substance, each variable taking the value 1 if that was the last drug used and 0 otherwise. ‘Employment’ was a binary variable taking the value 1 if employed, full- or part-time or studying, and 0 otherwise. ‘OAT’ was a binary variable taking the value 1 if the person stated that he or she was in OAT. Living situation was a binary variable taking the value 1 if the person stated that he or she had an ‘Independent living’ and 0 if living in an institution, instable housing situation, or homeless. ‘Daily drug use’ was a binary variable taking the value 1 if injecting drugs daily and 0 if less often. Standard errors were clustered at individual level to account for repeated measurements within the same individual.

We ran several model specifications, each extended with more variables. Model 1 consists of variables for age and gender, model 2 extend model 1 with a vector of health status, model 3 extend model 1 with a vector of substance use, and model 4 is the full model explained in ‘Eq. 1.’

We also assessed the estimated mean QALY at registration and at 6-, 12-, and 24-month follow-up to study if and how the HRQoL changed over time for persons enrolled in the NSP. The analysis was conducted as a pairwise testing of the mean QALY with a 95% confidence interval.

The study was performed in accordance with the Helsinki declaration and was approved by The Regional Ethical Review Board in Stockholm (Dnr: 2013/495–31/3).

## Results

In all, 550 PWID were included in the study. Descriptive statistics are presented in Table 1. The mean (SD) age of respondents was 41.7 (11.5) years, of which 20 percent were woman and 84 percent were born in Sweden (Table 1). Each respondent could answer the surveys up to four times (at enrollment in the study, at 6-, 12- and 24-month follow-up), resulting in a total of 1,145 observations. In 1,139 observations both surveys were answered at the same time or at least within a seven-day time frame (41.3% responded three times, 27.6% responded twice, 16.0% responded once, and 15.1% responded four times). A third (36%) of the respondent reported that they had an independent living, while the rest reported instable housing (e.g., temporary living with friends or family, in supportive housing, in institutions) or were homeless. Fourteen percent were employed full- or part-time or was studying. HCV was the most common infection in this population, with 66% having an active/viremic infection. A fourth, (24%), of the NEP population was also enrolled in OAT. A majority of the population were injecting daily, and the most common used drug was amphetamine. The sample in this study corresponds well to the larger cohorts of PWID in the Stockholm NSP, studied between 2013 and 2018. However, in this study there were larger proportions of participants using amphetamine and on OAT.

**Table 1:** Demographics of participants (all observations)

	Mean value *or* percentage	N
Age (mean (SD))	41.7 (11.5)	1140
Female gender (%)	20	1140
Born in Sweden (%)	84	1129
Independent living (%)	36	1104
Employed (%)	14	1104
Health status* (%)		
Hepatitis B positive	1	1101
Hepatitis C positive	66	1083
HIV positive	7	1126
Opioid agonist therapy (OAT) (%)	24	880
Injection drug use the past month (%)		1103
Daily	54	
A few times a month	41	
Less often than once a month	5	
Last drug used (%)		1127
Heroin	32	
Amphetamine	51	
Buprenorphine	6	
Methylphenidate	4	
Other	6	

*Health status indications are not mutually exclusive

### Data analysis Part 1: Analysis of instruments

A third (83 of 243) of all possible health states in EQ-5D were observed in the sample and 741 of 7500 (10%) possible health states in the SF-6D. 122 observations reported full health (11111) in the EQ-5D, compared to 25 observations (111111) in the SF-6D. No one reported the worst possible health state (33333) in EQ-5D, while six responses stated the worst health state (345555) in SF-6D.

**Fig. 1:**
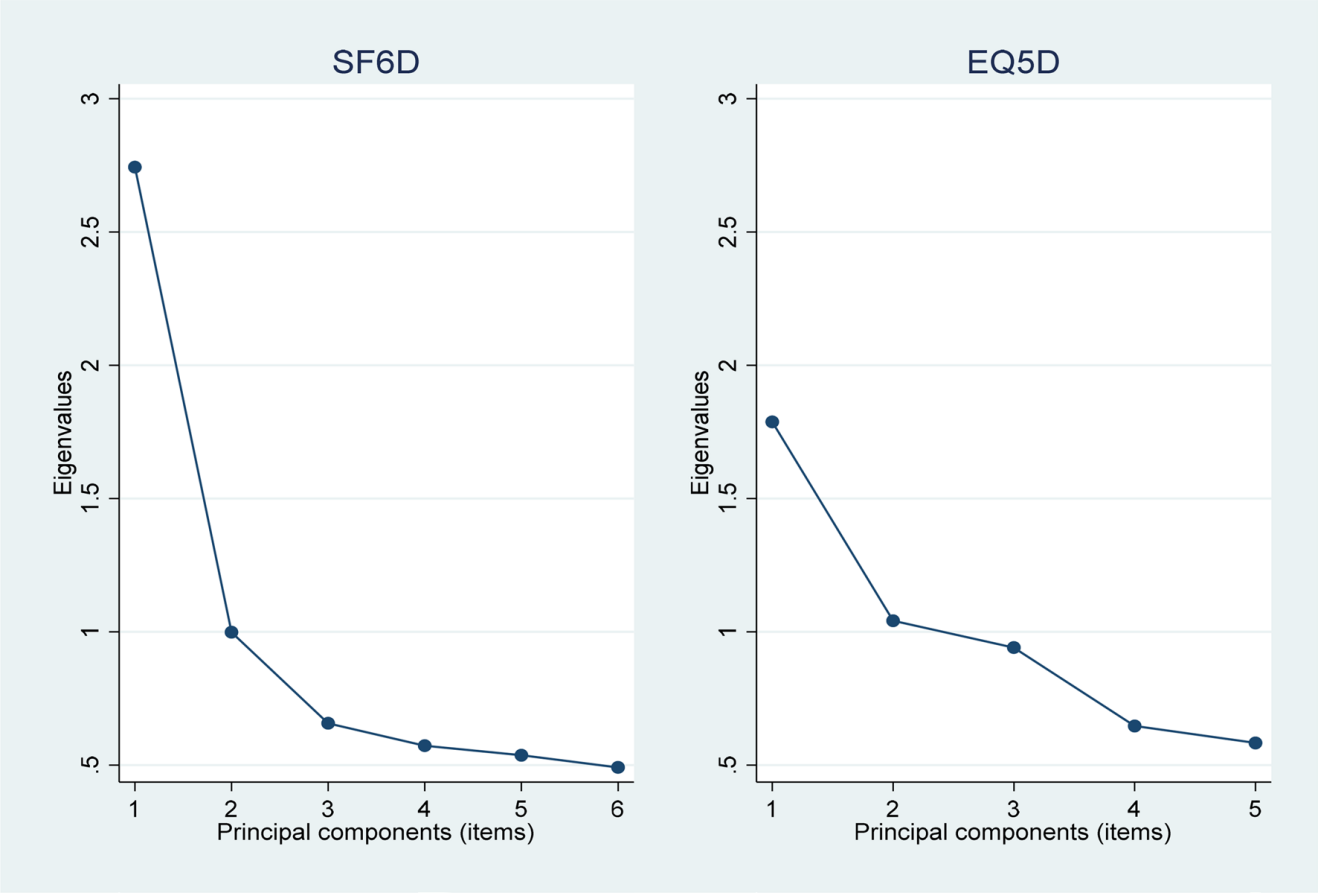
PCA scree plot for SF-6D and EQ-5D

The PCA for SF-6D demonstrated unidimensionality, with the first principal component capturing most of the variation in the data, the second component second most, and so on. For EQ-5D the explained variance was more evenly distributed among the principal components, which suggest that the instrument may measure multiple dimensions of health in contrast to SF-6D. In Fig. 1, the scree plot for EQ-5D and SF-6D show the level of variation each principal component captures from the data. Since differences in results from PCA for EQ-5D and SF-6D was relatively small, we conducted an IRT analysis on both instruments.

**Fig. 2:**
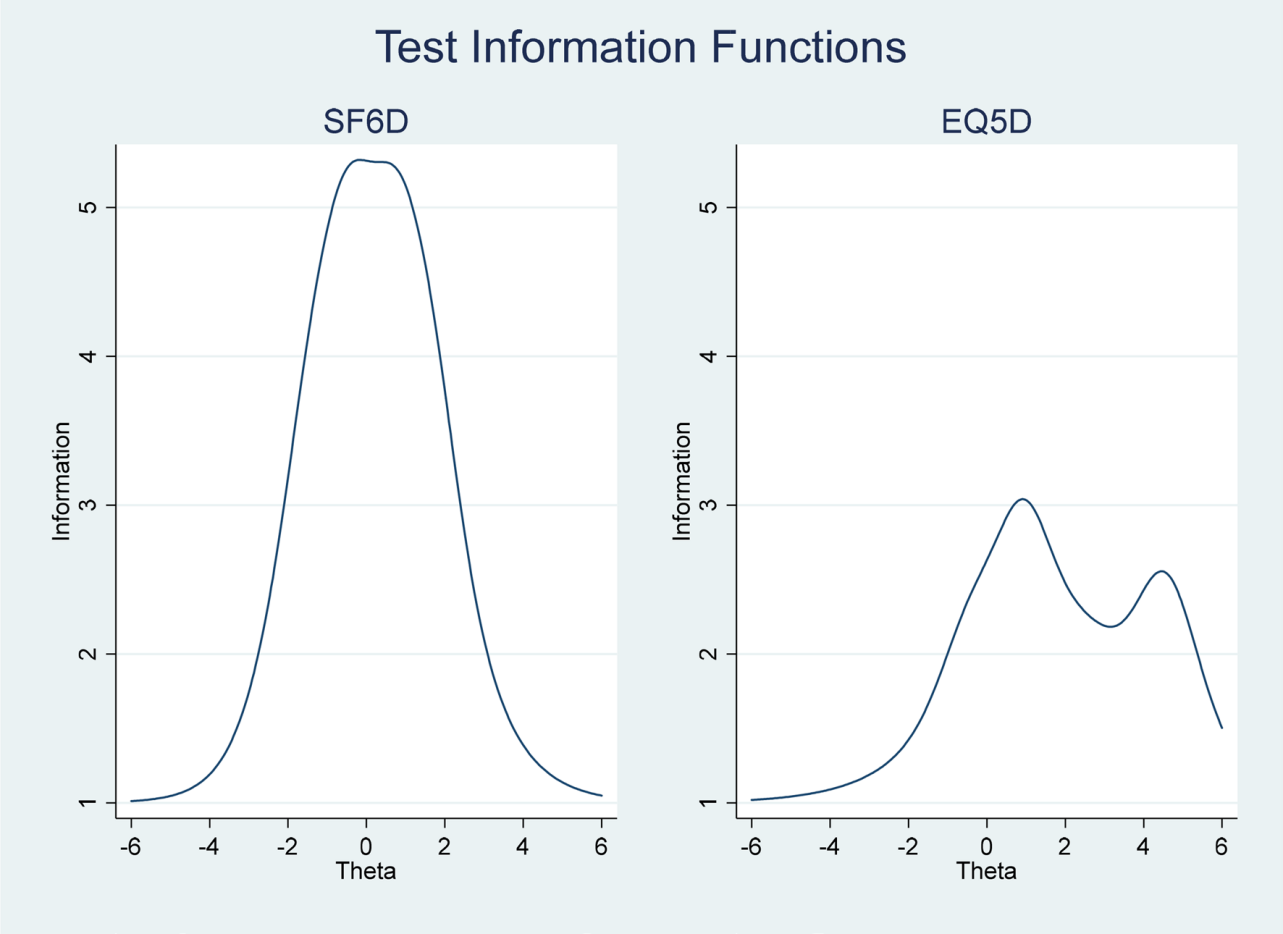
IRT: Test information function (Theta smaller than zero indicated health states better than average, while theta greater than zero equals health state worse than average)

In the IRT analysis, we assessed the item discrimination and the item difficulty for each item in both EQ-5D and SF-6D. Results (ordered according to the highest item discrimination parameter) show higher discrimination power on more items in SF-6D than in EQ-5D (Table 2). The item difficulty parameter shows that the order of health levels is reasonable for both questionnaires. However, more items in EQ-5D seems to be more suitable to detect persons with worse health status since several items (mobility, self-care, and usual activities) only have item difficulty coefficients above zero. In the test information functions, item discrimination and item difficulty parameters are combined for all items in the questionnaires, respectively (Fig. 2). Figure 2 confirms that EQ-5D was more suitable to detect persons with lower quality of life (more information when theta > 0), while SF-6D discriminates between persons regardless of their health status, with information normally distributed over the full range of HRQoL.

**Table 2:** Results from IRT analysis, item discrimination, and item difficulty parameters

EQ-5D	(a) Item discrimination	(b) Item difficulty
Pain/discomfort	1.75	
≥ 2		− 0.42
= 3		0.90
Mobility	1.74	
≥ 2		0.92
= 3		4.52
Self-care	1.56	
≥ 2		2.66
= 3		4.58
Anxiety/depression	0.84	
≥ 2		− 1.37
= 3		1.18
Usual activities	0.67	
≥ 2		0.45
= 3		4.03

SF-6D	(a) Item discrimination	(b) Item difficulty
Mental health	1.97	
≥ 2		− 1.48
≥ 3		− 0.37
≥ 4		0.95
= 5		1.69
Role limitation	1.90	
≥ 2		− 0.82
≥ 3		− 0.46
= 4		0.58
Social functioning	1.73	
≥ 2		− 0.63
≥ 3		− 0.20
≥ 4		0.51
= 5		1.19
Vitality	1.53	
≥ 2		− 2.07
≥ 3		− 1.04
≥ 4		0.22
= 5		1.55
Pain	1.03	
≥ 2		− 0.60
≥ 3		0.15
≥ 4		1.09
= 5		2.24
Physical functioning	1.03	
≥ 2		0.86
= 3		2.34

Since SF-6D performed better in both the PCA, demonstrating higher unidimensionality, and the IRT analysis, with the distribution of information being more evenly distributed over the full health spectrum, the analysis indicates that SF-6D is a better instrument to measure health in the PWID population compared to EQ-5D.

### Data analysis Part 2: Analysis of HRQoL

Since the IRT analysis used in part 1 showed that SF-6D was a better instrument to measure health in this population we used only the SF-6D to estimate QALY among PWID in part 2. In total, 547 respondents answered the SF-6D survey (three respondents were omitted from the full sample since they only responded to the EQ-5D survey).

In the regression analysis, independent of model specification, we found that QALY weight was significantly lower in all age groups compared to the reference group 60–69 years of age, ranging from about − 0.06 to − 0.011 (Table 3). Furthermore, we found that females had significantly lower QALY weights (-0.05) than males, which is a common relation also in the general population [33]. However, there were no significant effects on QALY weights of neither health status nor drug of choice in any of the specified models. Being employed, or studying, had a positive effect on the QALY weight, while enrolled in OAT or having an independent living had no significant effect on the QALY weight.

**Table 3:** Beta-coefficients from regression analysis, f(x) = QALY weight

	Model 1	Model 2	Model 3	Model 4
Age				
20–29	− 0.07^*^	− 0.06^*^	− 0.06^*^	− 0.08^*^
30–39	− 0.08^**^	− 0.07^*^	− 0.07^*^	− 0.09^*^
40–49	− 0.10^**^	− 0.09^**^	− 0.09^**^	− 0.11^**^
50–59	− 0.07^*^	− 0.07^*^	− 0.06^*^	− 0.07^*^
60–69 (ref. group)				
Female gender	− 0.05^***^	− 0.05^***^	− 0.05^***^	− 0.05^***^
Health status				
Hepatitis B positive		− 0.04		0.01
Hepatitis C positive		0.01		0.02
HIV positive		0.01		0.02
Substance use				
Heroin			0.01	− 0.00
Amphetamine			0.05^*^	0.04
Buprenorphine			− 0.00	− 0.00
Methylphenidate			0.05	0.03
Other (ref. group)				
Opioid agonist treatment (OAT)				− 0.02
Employed			0.04^**^	0.05^**^
Independent living				0.00
Daily injection drug use				− 0.01
Constant	0.77^***^	0.75^***^	0.72^***^	0.74^***^
Observations	1140	1055	1091	797
Adjusted *R*^2^	0.034	0.034	0.057	0.075

^*^ *p* < 0.05, ^**^ *p* < 0.01, ^***^ *p* < 0.001

From the pairwise mean testing, we found a significant improvement in mean QALY from registration to the 12-month follow-up, at a 5 percent significance level, which were maintained at the 24-month follow-up (Table 4). There was, however, no significant difference in mean between 6-, 12-, and 24-month follow-up.

**Table 4:** Pairwise mean testing of QALY weights between registration and follow-ups

Time of survey	Mean QALY (95% CI)
Registration	0.66 (0.65–0.67)
6 months	0.68 (0.65–0.72)
12 months	0.69 (0.68–0.71)
24 months	0.70 (0.68–0.72)

## Discussion

The aim of this study was twofold. First, we aimed to assess how well EQ-5D and SF-6D measures health-related quality of life in PWID. Secondly, we aimed to estimate HRQoL, in QALYs, in this population. Results from the first part of our analysis showed that SF-6D was a better-suited instrument to measure health in the PWID population. The IRT analysis showed that SF-6D, to a greater extent, discriminated between persons regardless of their health status, while EQ-5D was more suitable to detect persons with poorer health. This means that SF-6D was better suited to detect smaller changes in health, compared to EQ-5D, independent of if current health state was good or bad.

There could be several reasons for why SF-6D should be regarded as the preferred instrument among which we have identified two main reasons. First of all, SF-6D has an advantage in measuring mental health disorders compared to EQ-5D, as shown in previous studies [34, 35], which is an important aspect to consider when measuring health effects in PWID, where psychiatric comorbidity is highly prevalent [36–38].

Secondly, how questions and answers are framed in relation to the PWID population could have a great impact on the outcome. In addition to the mental health aspect, the health dimensions of SF-6D also cover a broader spectrum of health in general by including physical and social functioning, emotional role limitation, and vitality, which has been suggested as one of the main differences between SF-6D and EQ-5D overall [39]. For sub-populations of PWID, questions about mobility in EQ-5D, e.g., answered by “I am confined to bed” (the most severe level) could have a great effect on the outcome if answers are not applicable to current living situations, i.e., if respondents not necessarily have access to a stable housing (or a bed) themselves. In this aspect SF-6D answers are more general and, hence, easier to relate to independently of personal abilities.

Results from the second part of our analysis showed that average QALY weights in PWID was lower than in the general Swedish population [33]. For example, a male PWID aged 20–29 had a QALY weight of 0.66, compared to an average of 0.91 for a male of the same age in the general population [33]. These results show a great discrepancy in health between PWID and the general population. However, even though our result showed lower QALY weights in PWID compared to the general population, we could not state that there is a significant difference between these groups since this study was not conducted as a case–control study of health in different groups. Future studies should include people from both the general population and the PWID population to further study the differences in estimated QALY between populations. Although, if we believe that our results are comparable with previous studies of a general population, they suggest that the average QALY of a male PWID could be about 73% of the average QALY in a male in the general population. This could be compared to the previous study by Martin et al., which suggested that PWID had 84% of HRQoL of the general population.

In general, health is assumed to decrease with age, as noted in the study of the general Stockholm population by Burström et al. [33]. However, our results show that estimated QALY weights decreased by age and was lowest in persons aged 40–49 years and then, in contrast to the general population, increased with age. This result has not been shown previously and hence explanations are speculative. One possible explanation is that elder PWID may experience a more stable situation. In a previous study including partly overlapping participants, we found that reuse of unsterile equipment was less common among elder PWID [24]. This risk behavior may be correlated to health-related outcomes which thus also might be reflected in HRQoL measures. A similar reasoning may be applied regarding gender, where female participants reported lower HRQoL compared to males. It has been a consistent finding in research on PWID that women who inject drugs show a more severely impaired somatic, psychiatric, and social situation [40, 41], as well as injection-related behaviors [42]. It is therefore expected, but not previously shown, that women in this population show lower rates of HRQoL. A previous study by De Maeyer et al. indicated that OAT improves HRQoL, a result that was not confirmed in the present study [43]. In the study by De Maeyer et al., it was found that participants with a more stable situation regarding employment and living situation showed a higher HRQoL. In our study, OAT participants represented a minority of participants and generally, the rates of employment and stable living situation among these was low, providing possible explanations to why we did not find an effect of OAT on HRQoL.

Lastly, the results regarding improvement over time in HRQoL in a cohort of PWID has not previously been shown. The results are promising with regards to that participation in a NSP not only result in a reduction of risk behaviors for reuse of unsterile injection equipment [24], but also a general improvement in related subjectively experienced HRQoL. Future research should focus on what components more specifically are related to this improvement.

The most important contribution of this study may be that we now have more solid evidence regarding how to validly measure HRQoL in this population. Previous studies have failed to find a relation between HRQoL and health status as defined by a positive antibody status for HCV and self-reported status [19]. The present study corroborates these results, although our data relate to a confirmed viremic HCV status. The fact that HCV was so common in this population, with a prevalence over 60%, may reflect a limited concern and thus not having a major impact on quality of life. Still, it is important to stress that HRQoL was low among the participants, compared to the general population, and that lack of correlation between health status and HRQoL may reflect a saturated effect, leaving less room for variation related to HCV status. It is further important to note that in this study, health status was indicated by actual test results (positive, negative). It is probably important to also take into consideration that literacy of own health status may correlate with HRQoL [16], suggesting that future studies should include measures of both actual infection status and awareness of infection status among PWID.

### Strengths and limitations

Differences between our results and previous studies on the QALY weights in the general population may not be directly comparable since these studies did not aim at comparing populations. However, since the differences are quite large, this demonstrates the importance of using accurate methods and data when measuring health in PWID. Furthermore, the implications of using estimates from the general population in CEA of interventions aiming at PWID could have a great effect on the results.

## Conclusion

Access to reliable measures of health is equally important to such measures of costs in studies of cost effectiveness and hence, in health care decision-making and prioritization. It is therefore essential to have accurate methods that estimate health in different populations in a way that allow for comparison both within and between therapeutical areas. Results from this study increase knowledge about how suitable EQ-5D and SF-6D (through SF-12) are to measure health in the PWID population and highlight that fact that even though both EQ-5D and SF-6D are general questionnaires, they are more or less suitable for the PWID population. Furthermore, the results from our study contribute with actual levels of health in PWID and personal characteristics that effect the level of HRQoL. Reliable estimations of health are important not only to get an overall understanding of the current health status in a population but also to be able to use population relevant data to analyses of interventions that aim to increase health or prevent different risk behavior when prioritizing in the health care sector.
